# Characterization of *Triniti virus* supports its reclassification in the family *Peribunyaviridae*

**DOI:** 10.1099/jgv.0.001196

**Published:** 2018-12-14

**Authors:** Juliana Abreu Lima, Joaquim Pinto Nunes Neto, Karoline Silva Castro, Amélia Paes de Andrade Travassos da Rosa, Robert Tesh, Márcio Roberto Teixeira Nunes, Vsevolod Leonidovich Popov, Nikos Vasilakis, Hilda Guzman, Steven Widen, Sandro Patroca da Silva, Daniele Barbosa de Almeida Medeiros, Jedson Ferreira Cardoso, Lívia Carício Martins, Raimunda do Socorro da Silva Azevedo, Pedro Fernando da Costa Vasconcelos, Jannifer Oliveira Chiang

**Affiliations:** ^1^​ Department of Arbovirology and Hemorrhagic Fevers, Evandro Chagas Institute, Ministry of Health, BR 316, Km 07, S/N – 67030-000 – Levilândia - Ananindeua, Pará, Brazil; ^2^​ Department of Pathology, Institute of Human Infection and Immunity, University of Texas Medical Branch, 301 University Blvd, Galveston, TX 77555, USA; ^3^​ Center for Technological Innovation, Evandro Chagas Institute, Ministry of Health, BR 316, Km 07, S/N – 67030-000 – Levilândia - Ananindeua, Pará, Brazil; ^4^​ Department of Biochemistry and Molecular Biology, University of Texas Medical Branch, Galveston, TX 77555, USA

**Keywords:** *Peribunyaviridae*, *Triniti virus*, arbovirus, phylogenetic analysis, serologic tests, ultrastructure

## Abstract

*Triniti virus* (TNTV) has been isolated in Trinidad and Tobago and in Brazil. To date little is known about this virus, which is classified as an ungrouped virus within the family *Togaviridae*. Here, three isolates of TNTV were characterized both genetically and antigenically. The genome was shown to contain three RNA segments: small (S), medium (M) and large (L). Genome organization, protein sizes and protein motifs were similar to those of viruses in the genus *Orthobunyavirus*, family *Peribunyaviridae*. Antigenic reactivity revealed the three TNTV isolates to be closely related, but no serologic cross-reaction with other orthobunyaviruses. Morphological observation by transmission electron microscopy indicated that virus size and symmetry were compatible with those of viruses in the family *Peribunyaviridae.* Our serological, morphological and molecular results support the taxonomic reclassification of TNTV as a member of the genus *Orthobunyavirus*, family *Peribunyaviridae.*

## Introduction

**Fig. 1.**
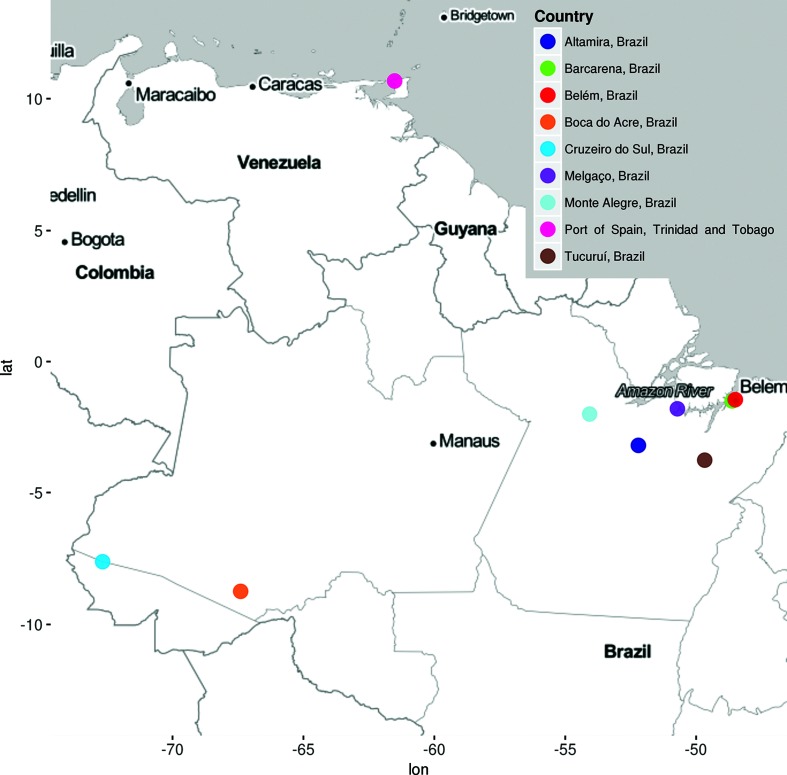
Map showing locations where *Triniti virus* isolates were obtained.

The prototype *Triniti virus* (TNTV), TRVL 7994, was isolated from a pool of 37 *Trichoprosopon* mosquitoes collected in Port of Spain, Trinidad, in 1955 (Fig. 1) [1]. The virus remained uncharacterized until 1964, when scientists at the Trinidad Regional Virus Laboratory (TRVL) demonstrated that TNTV was a new, enveloped and probably arthropod-borne virus [2]. In 1981, a study characterized some ungrouped viruses based on their morphological and physicochemical features; it concluded that TNTV was an enveloped spherical RNA virus about 65 nm in diameter, compatible with members of the family *Togaviridae*, and morphologically undifferentiated from *Sindbis virus* [3]. Since then, TNTV has been classified as an ungrouped virus in the family *Togaviridae* [4, 5].

The Brazilian TNTV prototype, BeAn 235467, was isolated from the blood of a rodent (*Dasyprocta aguti*) captured in Barcarena City, Pará State, in 1973 [6]. Subsequently, additional isolates of TNTV have been isolated in the Brazilian Amazon region from a variety of mosquito genera, including *Aedes*, Sabethes, *Wyeomyia* and *Anopheles* (Fig. 1) [7]. The present study sought to determine the taxonomic identity of TNTV by applying morphological, serological and genomic characterization of the virus prototype and two Brazilian isolates.

## Methods

### Viral isolates

The TNTV prototype was provided by the World Reference Center for Emerging Viruses and Arboviruses, University of Texas Medical Branch, Galveston, TX, USA. Two Brazilian TNTV isolates, BeAn 235 467 and BeAr 800584, were obtained from the virus collection of the Department of Arbovirology and Hemorrhagic Fevers, Evandro Chagas Institute, Ananindeua, Pará State, Brazil. BeAr 800 584 was isolated from *Aedes (Och) fulvus* mosquitoes collected in Belém City, Pará State, in 2014 (Table 1).

**Table 1.** Description of *Triniti virus* isolates used in this study

**Virus name**	**Strain number**	**Source**	**Locality**	**Date collected**	**Genbank accesion number (RNA segment)**
*Triniti virus*	TRVL 7994	*Trichoprosopon* spp.	Port of Spain, Trinidad and Tobago	1955	MG792213 to MG792215
*Triniti virus*	BeAn 235467	Rodent (agouti)	Barcarena – Pará, Brazil	1973	MG792207 to MG792209
*Triniti virus*	BeAr 800584	*Aedes (Och) fulvus*	Belém – Pará, Brazil	2014	MG792210 to MG792212

### Serological tests

To determine the antigenic relationship of TNTV with other New World orthobunyaviruses and phleboviruses, the TNTV antigen was used in complement fixation (CF) tests against hyperimmune mouse ascitic fluids prepared for each of the 66 viruses listed in Table S1 (available in the online version of this article). CF tests were carried out using a micro-technique, with two full units of complement [8]. The preparation of antigen and antisera was performed as described previously [8].

### Transmission electron microscopy

For structural analysis, ultra-thin sections of baby hamster kidney (BHK-21) cells infected with TNTV (TRVL 7994) were fixed for 1 h in a 2.5 v/w formaldehyde mixture prepared from paraformaldehyde powder and 0.1 v/v glutaraldehyde in 0.05 M cacodylate buffer, pH 7.3, to which 0.01 v/v picric acid and 0.03 w/w CaCl_2_ were added. The monolayer was washed in 0.1 M cacodylate buffer, cells were scraped off and the pellets were retained for further processing. The pellets were post-fixed in 1 % OsO_4_ in 0.1 M cacodylate buffer, pH 7.3, for 1 h, washed with distilled water and *en bloc* stained with 2 v/v aqueous uranyl acetate for 20 min at 60 °C. The pellets were dehydrated in ethanol, processed through propylene oxide and embedded in Poly/Bed 812 (Polysciences, Warrington, PA, USA). Ultra-thin sections were cut with a Leica EM UC7 ultramicrotome (Leica Microsystems, Buffalo Grove, IL, USA), stained with lead citrate and examined under a Philips 201 transmission electron microscope at 60 kV (Philips, Eindhoven, The Netherlands).

### Removal of contaminating host RNA and nucleic acid isolation

Supernatant culture fluids from African green monkey kidney (Vero) or BHK-21 cells infected with the three TNTV isolates were collected between 3 and 5 days post-infection, when 80–90 % cytopathic effect was observed. They were centrifuged for 10 min at 2000 rpm (700***g***) and then for 5 min at 4000 rpm (2800***g***), followed by filtration through a 0.2 µm sterile filter and polyethylene glycol precipitation to enrich for virus particles. Subsequently, the virus pellets were resuspended in 250 µl phosphate-buffered saline, and 25 µl RNase A (final concentration 2 mg ml^−1^) was added to digest cellular RNAs. Next, viral RNA was extracted using the QIAamp Viral RNA Mini Kit (Qiagen, Hilden, Germany), according to the manufacturer’s instructions.

### Nucleotide sequencing

Complementary DNA (cDNA) synthesis was performed using the cDNA Synthesis System Kit (Roche Diagnostics, Rotkreuz, Switzerland) and 400 µM random primers (Roche Diagnostics). The reaction solution was purified with the Agencourt AMPure XP Reagent (Beckman Coulter, LaBrea, CA, USA). The cDNA libraries of the Brazilian TNTV isolates were subjected to semiconductor sequencing [9] (Ion Torrent PGM; ThermoFisher Scientific, Waltham, MA, USA) at the genomic core facility of the Center for Technological Innovation, Evandro Chagas Institute. The cDNA library of the TNTV prototype was sequenced on a HiSeq 2500 System (Illumina, San Diego, CA, USA) at the genomic core facility of the University of Texas Medical Branch. The 5′ and 3′ termini of non-coding regions (NCR) from the Brazilian isolates were amplified by 5′/3′ RACE kits following the manufacturer’s instructions (Roche, Basel, Switzerland) using Sanger sequencing method [10].

### Genome assembly and phylogenetic analysis

Sequence assembly was carried out using the *de novo* assembler within the MIRA 4.0 program [11]. Inspection and annotations of putative open reading frames (ORFs) were performed using Geneious 9.1.6 software (Biomatters, Auckland, New Zealand). The final versions of all segments were compared with other sequences available in the NCBI (http://www.ncbi.nlm.nih.gov/) using Basic Local Aligning Search Tool (BLASTx).

Multiple sequence alignment was performed using the PROMALS3D (http://prodata.swmed.edu/promals3d/promals3d.php) open source program [12]. The dataset used to reconstruct the phylogenetic trees consisted of amino acid sequences obtained for each genome segment of representative members of the genus *Orthobunyavirus*. A maximum likelihood tree was used to reconstruct the TNTV phylogenetic relationship [13] using RAxML 8.2.9 software [14]. Bootstrap analysis was conducted over 1000 replicates [15].

Confidence values used as criteria for group inclusion or exclusion were calculated based on the mean of amino acid sequence divergence within and between groups, in line with classical taxonomic classification within the genus *Orthobunyavirus*. A paired *t*-test was applied and group clustering was considered statistically significant at *P*-values <0.05.

### Polyprotein analysis

Prediction of N-glycosylation sites was carried out using the NetNGlyc 1.0 open source program (http://www.cbs.dtu.dk/services/NetNglyc/). The SignalP 4.1 server (http://www.cbs.dtu.dk/services/SignalP) was used to predict the presence and location of signal peptide cleavage sites in amino acid sequences. Both analyses were performed using the amino acid sequence of the M polyprotein.

### Evaluation of genetic reassortment

Natural genetic reassortment was evaluated at nucleotide (nt) level using concatenated ORFs for all three RNA segments (SRNA, MRNA, LRNA). To evaluate genomic shifting, since this is a common process within the *Peribunyaviridae* family, multiple sequence alignment was carried out first with MAFFT 7 [16] and then with SimPlot 3.5.1 software [17]. Values of permutation trees were assigned as percentages. Genetic reassortment was considered when the percentage of permutation trees was higher than 90 % across the entire genome segment.

## Results

### Antigenic relationships

CF tests confirmed that, based on antigenic reactivity, the three TNTV isolates were closely related to each other (Table 2). In contrast, no serologic cross-reactions were observed between the TNTV antigen and antibodies prepared against any of the 66 New World orthobunyaviruses and phleboviruses tested (Table 2).

**Table 2.** Serological results of complement fixation tests with three *Trinity virus* isolates and representative species of antigenic groups of the families *Peribunyaviridae* and *Phenuiviridae*

Virus**	Antibody*		
	TRVL 7994	BEAn 235467	BEAr 800584
TRVL 7994	512/32	256/8	nt
BeAn 235467	512/32	512/32	128/4
BeAr 800584	nt	128/4	128/8
*Tacaiuma virus*	0/0	0/0	0/0
*Caraparú virus*	0/0	0/0	0/0
*Guaroa virus*	0/0	0/0	0/0
*Maguari virus*	0/0	0/0	0/0
*Guamá virus*	0/0	0/0	0/0
*Oropouche virus*	0/0	0/0	0/0
*Gamboa virus*	0/0	0/0	0/0
*Turlock virus*	0/0	0/0	0/0
*Capim virus*	0/0	0/0	0/0
*Icoaraci virus*	0/0	0/0	0/0

*Reciprocal of highest antibody titre/reciprocal of highest antigen titre

**One representative species from each antigenic group. The results for other viruses are given in the text.

NT=not tested due to absence of antigens and homologous serum.

### Ultrastructural studies

**Fig. 2.**
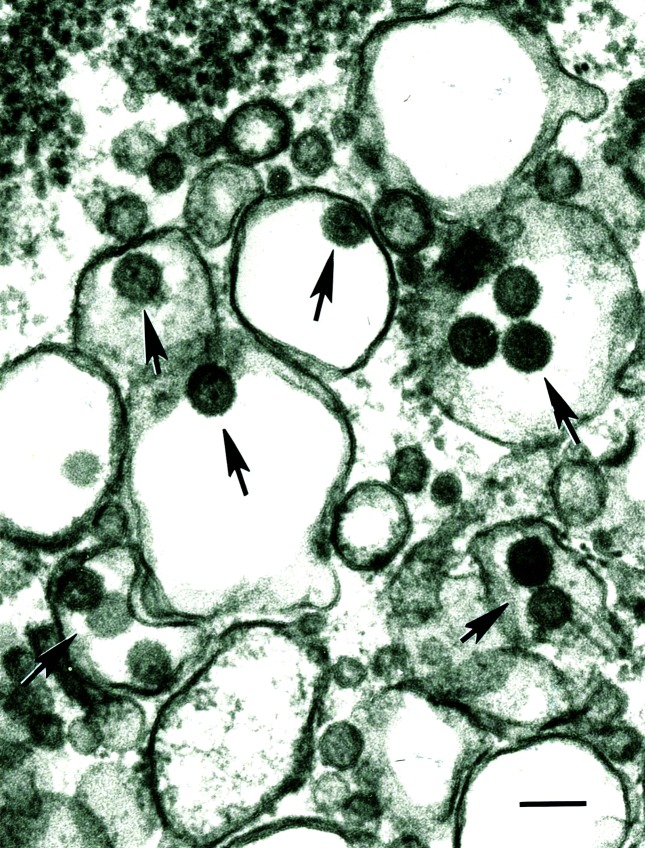
Transmission electron microscopy of *Triniti virus*. Shown is an ultra-thin section of BHK-21 cells infected with the TNTV TRVL 7994 isolate. Arrows indicate viral particles. Bar, 100 nm.

Transmission electron microscopy of ultra-thin sections revealed the existence of spherical viral particles, about 80 nm in diameter, inside vacuoles of BHK-21 cells infected with TNTV (TRVL 7994) (Fig. 2).

### Full-length sequencing, genome organization and NCRs at 3′ and 5′ termini

**Fig. 3.**
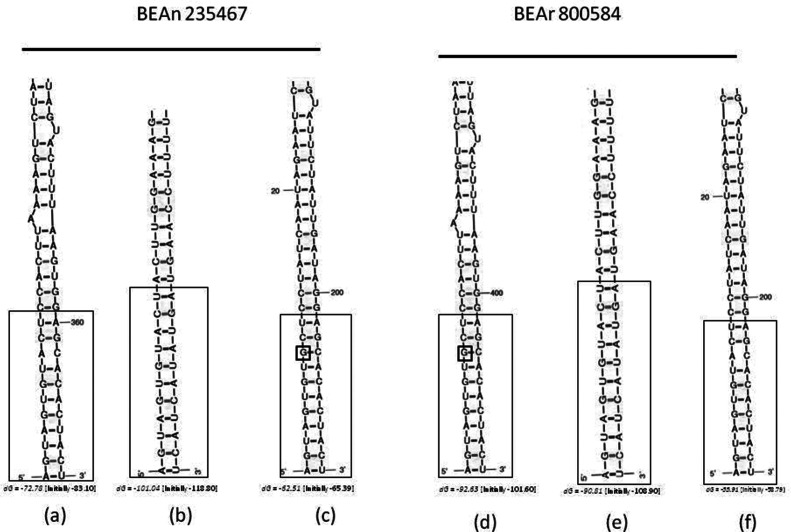
Folded structure of the 5′ and 3′ non-coding regions in SRNA (a and d); MRNA (b and e) and LRNA (c and f) for *Triniti* Brazilian isolates BeAn 235467 and BeAr 800584. dG corresponds to the energy level used to stabilize the structure. Highly conserved nucleotide sequences are enclosed by large boxes. Small boxes with a G represent nucleotide mismatch.

Complete genome sequences were obtained for TNTV BeAn 235467 (SRNA, 1089 nt; MRNA, 4863 nt; LRNA, 6907 nt) and BeAr 800584 (SRNA, 1094 nt; MRNA, 4863 nt; LRNA, 6907 nt), whereas a nearly complete genome was recovered for TRVL 7994 (SRNA, 1088 nt; MRNA, 4737 nt; LRNA, 6868 nt). Conserved terminal sequences were observed in the complete sequences of SRNA (5′-AGTAGTGTACTCCACTTAAA……TTTAAGTGGAGCACACTACT-3′), MRNA (5′-AGTAGTGTACTACTTGGAAA……TTTCCAAGTAGTATACTACT-3′), and LRNA (5′-AGTAGTGTGCTCCTATCAAT………ATTGATAGGAGCACACTACT-3′). SRNA, MRNA and LRNA folded structures are shown in Fig. 3. The genomes consisted of single-stranded, tri-segmented, negative-sense RNA molecules.

he Brazilian strains of *Triniti virus* showed high identity between each other to all segments in comparison with the Trinidad strain (Table 3). Furthermore, BlastX analysis identified the S segment to be most closely related to *Lukuni virus* (E-value, 5e-80; nt identity, 51 %), the M segment to *Tacaiuma virus* (E-value, 0.0; nt identity, 47 %) and the L segment to *Anopheles A virus* (E-value, 0.0; nt identity, 64 %). Table S2 summarizes the basic characteristics of each TNTV genome segment.

**Table 3.** Matrix of amino acid (green) and nucleotide (blue) identity between different *Triniti virus* isolates

		SRNA			MRNA			LRNA		
	Isolate	1	2	3	1	2	3	1	2	3
1	TRVL 7994		98.0	97.6		95.4	94.9		97.6	97.1
2	BeAr 800584	86.1		99.6	81.7		98.9	83.2		99.2
3	BeAn 235467	85.5	91.3		81.6	94.8		83.1	91.6	

### S segment

The SRNA of the three TNTV isolates contained only one ORF (747 nt), which encoded the nucleocapside (N) protein comprising 248 aa (28.5 kDa) and two Cys residues. The non-structural (NS) protein was not present. Analysis of residues involved in packaging of orthobunyavirus ribonucleoprotein (P_125_, G_131_, Y_158_ and I_231_), RNA synthesis (F_17_, F_144_, L_160_, Y_176_, L_177_, K_179_, Y_185_, W_193_, W_213_ and F_225_) and RNA binding (R_40_, R_94_, and K_50_) indicated that, except for I_231_L and W_213_M (positions relative to *Bunyamwera virus*), TNTV was rather well conserved.

### M segment

The MRNA of TNTV comprised a coding region of 4335 nt that encoded a polyprotein (Gn-NSm-Gc) of 1444 aa (163.3 kDa). This was post-translationally cleaved into three proteins: the glycoprotein Gn of 306 aa (34.8 kDa), the glycoprotein Gc of 966 aa (109.2 kDa) and the non-structural NSm protein of 172 aa (19.2 kDa).

The cleavage sites between Gn-NSm and NSm-Gc were located at positions SAR/YM and AGF/GC, respectively. The MRNA encoded for 72 Cys residues and five probable N-linked glycosylation sites (positions 696, 714, 738, 1017 and 1179). A number of features appeared conserved in the M segment of TNTV. These include the zinc-finger motif, which enables binding between the cytoplasmic portion of Gn and the N protein during viral replication; the *Orthobunyavirus*-specific fusion peptide WGCEExGCLAxxxGCV(F/Y)GSCQDXI (except at positions V_1068_I, S_1086_Q and Q_1078_K); and the A_475_ cleavage site between NSm-Gc, described for members of the California group (*Orthobunyavirus*, *Peribunyaviridae*).

### L segment

RNA-dependent RNA polymerase (RdRp) of TNTV isolates was encoded by 6735 nt, which produced a 2244 aa protein (261.1 kDa). Amino acid alignment revealed the presence of four functional regions on the RdRp protein (I, II, III and IV). Based on alignment of the TNTV TRVL 7994 sequence, regions I and II were located in the central N-terminal part of RdRp and were characterized by the conserved residues P_78_D_79_ (region I) and R_653_Y_654_ (region II).

Region III was located between positions 1020 and 1212 of the TNTV L protein. It contained the following conserved motifs at the respective positions: A (1032 to 1049), B (1117 to 1138), C (1157 to 1171) and D (1201 to 1212). Additionally, within region III, it was possible to detect the conserved pre-motif A (951 to 980) and motif E (1213 to 1223) described previously [18]. Finally, region IV, located at positions 1236 to 1251 and 1288 to 1304, was also conserved in TNTV.

### Phylogenetic analysis and group definition

**Fig. 4.**
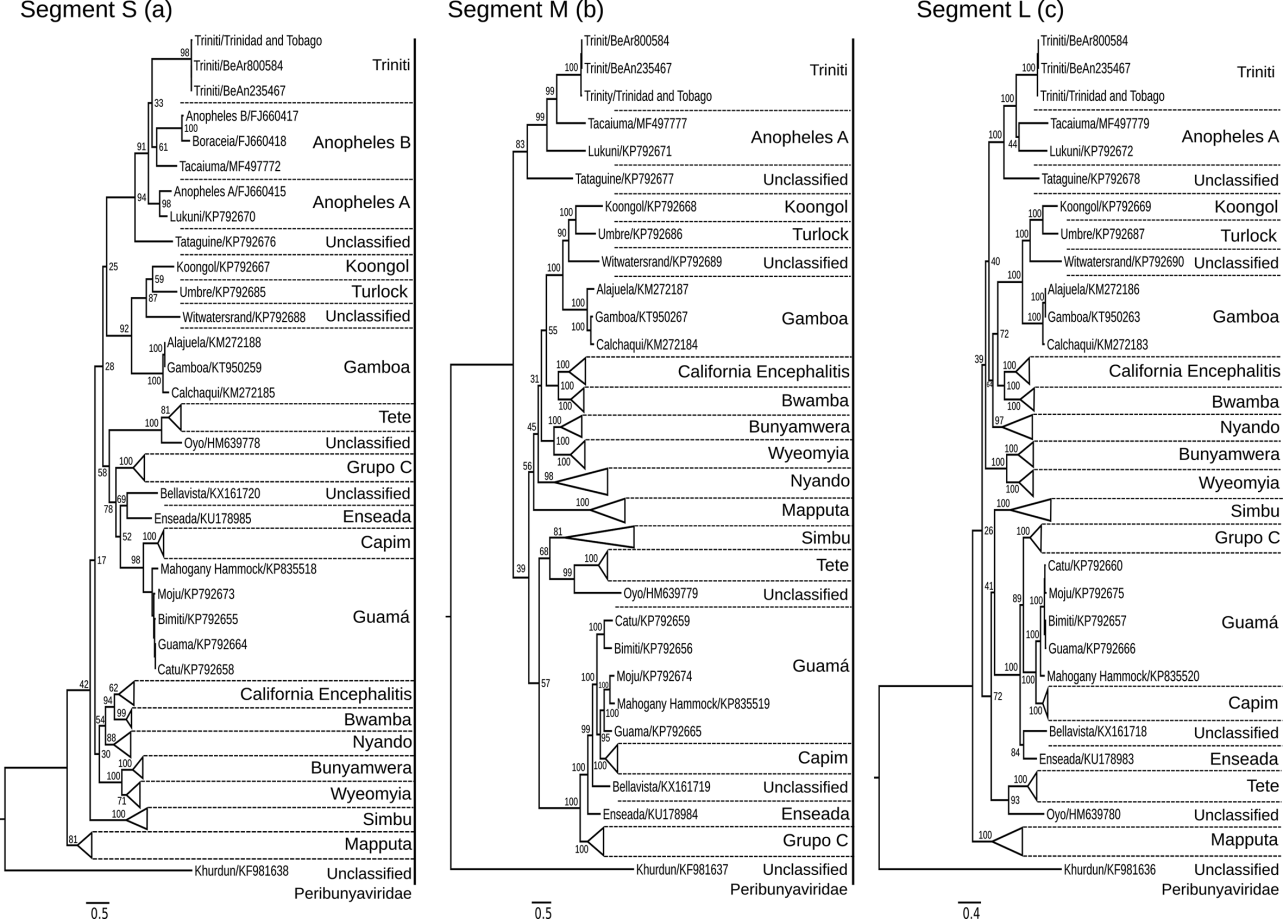
Maximum likelihood phylogenetic trees of three *Triniti virus* isolates: S segment (a), M segment (b) and L segment (c).

Regardless of RNA segment, phylogenetic analysis of nucleotide and amino acid sequences showed similar topologies for the N gene, M polyprotein and RdRp. This suggested that the three TNTV isolates belonged to a monophyletic group (bootstrap 1000, *P*-value <0.05 based on a paired *t*-test). TNTV isolates were most closely related to *Tacaiuma virus*, *Anopheles A* group and *Tataguine virus* (Fig. 4a–c).

### Reassortment analysis

SimPlot analysis failed to provide evidence of any natural reassortment events between the genomic segments of TNTV and other closely related orthobunyaviruses.

## Discussion

Despite its isolation more than 60 years ago, relatively little information is available about TNTV. The first report on the virus, in 1964, described some of its physical and biological properties and concluded that TNTV was an enveloped RNA virus, probably arthropod-borne [2]. Results of a second morphological and physicochemical study, published in 1981, concluded that TNTV was an enveloped spherical RNA virus compatible with the *Togaviridae* family [3]. At the eighth International Committee on Taxonomy of Viruses Report on Virus Taxonomy, TNTV was listed as an unassigned virus among *Togaviridae* [4]. TNTV was not included in the ninth Report on Virus Taxonomy [19], and the Arbovirus Catalog still lists TNTV as an ungrouped togavirus [5].

Our analysis of the genome organization of TNTV revealed three RNA segments (SRNA, MRNA and LRNA), encoding the N, Gn-NSm-Gc and L proteins, respectively. This genome organization pattern, as well as protein sizes and motifs, is typically found in members of the recently established order *Bunyavirales*, family *Peribunyaviridae* [20]. Members of the family *Peribunyaviridae*, more specifically in the genus *Orthobunyavirus*, possess a tripartite genome, composed by single-stranded, negative-sense RNA segments, namely SRNA, MRNA and LRNA. SRNA, in general, encodes a nucleocapside protein (N protein) and a non-structural protein S (NSs). The M segment encodes a large and unique polyprotein that after cleavage gives rise to the glycoproteins Gn and Gc, as well as to the non-structural M protein (NSm). For the SRNA, a large and unique polyprotein is encoded and corresponds to the polymerase protein. The 3′ and 5′ terminal regions are found and correspond in general to 15 highly conserved and complementary sequences, 5′ AGT AGT GTA CTC CAC …… TGG GAG CAC ACT ACT 3′ [20]

The size of the three RNA segments, the presence of highly conserved nucleotides at the 3′ and 5′ complementary terminal sequences, as well as the Gn-NSm-Gc protein organization of the MRNA ORF are compatible with viruses in the genus *Orthobunyavirus* [20]. Interestingly, the SRNA ORF of TNTV does not encode a NS protein, as observed in other orthobunyaviruses, especially *Tataguine*, *Tacaiuma* and *Anopheles A* and *B* viruses. The NS protein is an important virulence factor because it acts as an interferon antagonist and thus helps viral particles escape the host immune system. However, it is not essential for viral replication [21–23]. TNTV appears to possess various conserved features previously identified in other orthobunyaviruses. These include the Cys residues on the M polyprotein; five potential N-linked glycosylation sites on Gn, including conserved sites at positions N_60_ and N_1169_; and functional motifs, such as zinc-finger and fusion peptide on Gc, as well as RdRp on the L protein [22].

A phylogenetic analysis of the three RNA segments performed at amino acid level demonstrated a well-defined clade grouping of the three TNTV isolates. Supported by a high bootstrap value, analysis of the S segment indicates that TNTV shares the same ancestor as *Tataguine virus*, *Tacaiuma virus* and members of the *Anopheles A* and *B* group viruses. Even though the RNA sequences of the M and L segments of the *Anopheles B* group viruses are not available, TNTV shows high divergence from the *Anopheles A* group (*Anopheles A virus* and *Lukuni virus*) and *Tataguine virus* (Fig. 4).

Regardless of the RNA segment, the paired *t*-test revealed a statistically significant *P*-value (*P*<0.05), suggesting that TNTV belongs to a specific group distinct from any other. Backed by a consistent statistical analysis (bootstrap and *P*-values), these results suggest that TNTV constitutes a new phylogenetic cluster. Furthermore, no evidence of natural reassortment involving the TNTV isolates and other orthobunyaviruses could be detected.

Results from serological tests indicate that the three TNTV isolates are closely related and form a single antigenic group (Table 2); however, they are unrelated to 66 other New World viruses of the *Peribunyaviridae* and *Phenuviridae* families. Likewise, transmission electron microscopy on ultra-thin sections of BHK-21 cells infected with TNTV demonstrated the presence of virions with morphological characteristics and size similar to those of viruses in the family *Peribunyaviridae*.

To date, all TNTV isolates have been recovered from mosquitoes and wild rodents. In the initial description of the virus, Spence and co-workers examined 95 human sera from two communities in eastern Trinidad and from TRVL staff. Using a newborn mouse neutralization test, they found that seven individuals (7.4 %) had protective antibodies against the virus. Interestingly, one individual (a TRVL employee) demonstrated seroconversion to the virus in two baseline sera collected six years apart. These data demonstrate the potential of TNTV to infect humans; however, only additional serological studies will confirm the pathogenesis of the virus and the actual threat that it may pose to public and veterinary health.

In summary, our analyses support the reclassification of TNTV into the genus *Orthobunyavirus*, family *Peribunyaviridae* and the possibility that it constitutes a new species in this genus. Additional molecular, serological and pathological studies will clarify the potential role of TNTV in human infections.

## Supplementary Data

Supplementary File 1
