# Supplementary material for: Characterization of Triniti virus supports its reclassification in the family Peribunyaviridae
Source: J Gen Virol. 2018 Dec 14;100(2):137–44. doi: 10.1099/jgv.0.001196 (PMC7011695; doi:10.1099/jgv.0.001196)
Supplement: Supplementary File 1 [file jgv-100-137-s001.pdf]

Supplementary Table 1: Description of viruses from families *Peribunyaviridae* and *Phenuiviridae* used in the complement fixation tests with *Triniti virus* isolates, evaluating the serologic relationship between these viruses.

FAMILY	ANTIGENIC GROUP	VIRUS
PERIBUNYAVIRIDAE	**Anopheles A**	Arumateua, Caraipé, Lukini, Tacaiuma, Trombetas, Tucuruí,
	**Group C**	Apeú, Caraparú, Itaquí, Marituba, Murutucú, Nepuyo, Oriboca, Caraparú-like,
	**Califórnia**	Guaroa, Melao, Serra do navio
	**Bunyamwera**	Iaco, Kairi, Macauã, Maguari, Sororoca, Tucunduba, Taiassui, Xingu
	**Guamá**	Ananindeua, Bimiti, Catú, Guamá, Mirim, Mojú, Timboteua
	**Simbu**	Jatobal, Oropouche, Utinga
	**Gamboa**	Gamboa-like
	**Turlock**	Turlock
	**Capim**	Acará, Benevides, Benfica, Bush Bush, Capim, Guajará, Moriche
PHENUIVIRIDAE	**Phlebotomus fever**	Alenquer, Ambé, Anhanga, Ariquemes, Belterra, Bujarú, Candirú, Icoaraci, Itaituba, Itaporanga, Jacundá, Joá, Morumbi, Mucura, Munguba, Oriximiná, Salobo, Serra Norte, Tapará, Turuna, Uriurana, Urucuri

Supplementary table 2: Characteristics for S, M and L genome segments of *Triniti virus*. Analysis was performed using sense 5’ to 3’.

ISOLATE	5' NCR	TERMINAL SEQUENCE	N	M POLYPROTEIN			L POLYPROTEIN	3' NCR	TERMINAL SEQUENCE	Total
				Gn	Nsm	Gc				
**BeAN235467**	42nt	AGTAGTGTACTCCACTTAAA…	747 nt 248aa (28.5kDa)	NA	NA	NA	NA	300 nt	...TTTAAGTGGAGCACACTACT	1.089 nt
**BeAR800584**	42nt	AGTAGTGTACTCCACTTAAA…	747 nt 248aa (28.5kDa)	NA	NA	NA	NA	305 nt	...TTTAAGTGGAGCACACTACT	1.094 nt
**TVRL 7994**	42nt	AGTAGTGTACTCCACTTAAA…	747 nt 248aa (28.5kDa)	NA	NA	NA	NA	299 nt	...TTTAAGTGGAGCACACTACT	1.088 nt
				ORF: 4.335 nt/1.444 aa/163.3 kDa						
**BeAN235467**	59nt	AGTAGTGTACTACTTGGAAA…	NA	284 aa (32.3 kDa)	167aa (18.8 kDa)	861aa (97.5 kDa)	NA	469 nt	...TTTCCAAGTAGTATACTACT	4.863 nt
**BeAR800584**	59nt	AGTAGTGTACTACTTGGAAA…	NA	284 aa (32.3 kDa)	167aa (18.8 kDa)	861aa (97.5 kDa)	NA	469 nt	...TTTCCAAGTAGTATACTACT	4.863 nt
**TVRL 7994**	59nt	AGTAGTGTACTACTTGGAAA…	NA	284 aa (32.3 kDa)	284 aa (32.3 kDa)	284 aa (32.3 kDa)	NA	Not complete		4.737 nt
**BeAN235467**	33 nt	AGTAGTGTGCTCCTATCAAT…	NA	NA	NA	NA	6.732nt 2.243aa (261 kDa)	142 nt	...ATTGATAGGAGCACACTACT	6.907 nt
**BeAR800584**	33 nt	AGTAGTGTGCTCCTATCAAT…	NA	NA	NA	NA	6.732nt 2.243aa (261 kDa)	142 nt	...ATTGATAGGAGCACACTACT	6.907 nt
**TVRL 7994**	not complete	-------------GTGCTCCTATCAAT...	NA	NA	NA	NA	6.732nt 2.243aa (261 kDa)	Not complete	...ATTGATAGGAGCAC------------	6.868nt

*NCR: Non coding region; NA: Not applicable; ORF: Open reading frame; nt: nucleotide; aa: Amino acidy;
